# Pregnancy-Associated Cardiomyopathy in a Late-Diagnosed Partial Hydatidiform Mole: A Case Report

**DOI:** 10.1155/crog/2266206

**Published:** 2025-11-14

**Authors:** S. Uehlein, T. König, D. Berliner, H. Freitag, J. Bauersachs, P. Hillemanns, L. Brodowski, L. Steinkasserer

**Affiliations:** ^1^Department of Cardiology and Angiology, Medical School Hannover, MHH, Hannover, Germany; ^2^Institute for Pathology, Medical School Hannover, MHH, Hannover, Germany; ^3^Department of Gynecology and Obstetrics, Division for Obstetrics, Gynecology and Reproductive medicine, Medical School Hannover, MHH, Hannover, Germany

**Keywords:** abortion, bromocriptine, cardiomegaly, cardiomyopathy, case report, hCG, hydatidiform mole, hyperthyroidism, IUFD, NT-proBNP, pregnancy, prolactin, TSH

## Abstract

**Background:**

The hydatidiform mole presents as either a complete or partial mole. They are differentiated by morphology, histopathology, karyotype, and the risk of malignancy. Partial hydatidiform moles are the only type of trophoblastic gestational disease associated with the presence of a fetus (with or without positive cardiac response). However, early intrauterine fetal death often occurs with triploidy. Therefore, a partial hydatidiform mole is often misdiagnosed as an incomplete abortion. This case is unique due to the rare occurrence of a partial hydatidiform mole complicated by severe pregnancy-associated cardiomyopathy. To date, only five similar cases have been reported, all describing cardiorespiratory symptoms from left ventricular dysfunction in previously healthy women around abortion induction.

**Case Report:**

We present the case of a 19-year-old 1 gravida, 0 para with partial hydatidiform mole and late miscarriage in the 18th week of pregnancy. Abortion induction occurred, followed by severe maternal cardiac complications. The patient developed a pregnancy-associated cardiomyopathy with acute heart failure on the basis of a newly severely restricted biventricular function with dilatation and secondary mitral valve insufficiency, congestion and forward failure led to congestive pneumonia, acute renal failure, and metabolic acidosis. After histological examination of the fetal tissue, a partial mole can be assumed on the basis of the histological findings, immunohistochemistry, and the trisomy of the X chromosome detected by fluorescence in situ hybridization.

**Conclusion:**

In case of a hydatidiform mole, an early diagnosis is essential to prevent serious complications during medical course. Particularly, if cardiac symptoms occur, early diagnosis should be carried out. Close cardiological and gynecological follow-up must be carried out to prevent late complications.

## 1. Introduction

Gestational trophoblastic disease (GTD) originates in the placenta. The primary localization is the uterus, although peripheral metastases are possible. Hydatidiform mole is one of the forms of GTD. The pathogenesis of GTD is complex, as the tumor does not develop from maternal tissue but from pregnancy tissue [1]. The hydatidiform mole can exist as a complete or partial form. The difference between the two forms is the chromosome pattern, the histopathology, and the clinical presentation. The prognosis depends on the entity [2–4]. Molar pregnancies may be precancerous. If left untreated or if treated ineffectively, hydatidiform moles can progress to invasive disease. Gestational trophoblastic neoplasia (GTN) includes invasive mole, chorionic carcinoma, placental site trophoblastic tumor (PSTT), and epithelioid trophoblastic tumor (ETT). The hydatidiform mole originates in the villous trophoblasts. Chorionic villi with trophoblast hyperplasia as a result of overexpression of the paternal genes are the consequence [5, 6]. The difference between the two hydatidiform moles can be determined by analyzing the chromosome set. Complete hydatidiform moles are diploid, whereas partial moles are triploid. Complete hydatidiform moles are more likely to progress to malignant disease than partial hydatidiform mole [1, 7]. Risk factors for developing a hydatidiform mole include a previous hydatidiform mole (increased risk of about 1% to 1.5%—about 10 to 15 times the risk in the general population) [8–10] and very low or very high maternal age (≤ 15 and > 35 years) [9, 11]. The risk of developing a hydatidiform mole is significantly higher in women with a history of spontaneous abortion or infertility. Compared to patients with no history of miscarriage, the risk of a complete (3.1-fold) and partial (1.9-fold) mole is significantly higher in patients with a history of two or more miscarriages. Difficulty conceiving or infertility is associated with an odds ratio of 2.4 and 3.2 for a complete and partial mole, respectively [12–14].

Clinical symptoms of hydatidiform mole pregnancy are usually complications in early pregnancy. These usually include vaginal bleeding or hyperemesis gravidarum [7, 15, 16]. However, a hydatidiform mole is often only discovered after an abortion, as part of the pathological examination of the aborted material [17]. An abnormally high serum hCG level or ultrasound findings may indicate an early stage hydatidiform mole. A Swedish study by Joneborg et al. showed that about 40% of all patients with complete hydatidiform mole were asymptomatic [18]. Apparently, the availability of both ultrasound and sensitive quantitative measurement of serum hCG levels has led to earlier diagnosis of complete mole [19–22]. Before this, patients with hydatidiform moles mainly presented with late symptoms such as hyperthyroidism and pre-eclampsia. However, early detection does not reduce the risk of developing postmolar GTN [15, 23]. The most common early symptoms of hydatidiform moles are vaginal bleeding, abdominal or pelvic pain and tenderness, enlarged uterus, and hyperemesis gravidarum [15]. Late symptoms include ovarian theca-lutein cysts, hyperthyroidism, and early onset of pre-eclampsia. All these symptoms are associated with massively elevated hCG levels. Hyperthyroidism often develops with an hCG value of > 100,000 mIU/mL over several weeks. Clinical correlates for hyperthyroidism are tremor, tachycardia, and warm skin [24–26]. Histological confirmation is the gold standard of diagnosis for hydatidiform moles. Complete and partial moles are differentiated on the basis of histopathology, karyotype (complete hydatidiform moles are diploid, partial moles are triploid), and the presence of a fetus (a fetus is only present in partial hydatidiform moles) [27–29]. The standard treatment for hydatidiform mole is uterine evacuation via suction curettage, regardless of childbearing status. Hysterectomy may be considered in selected high-risk cases—for example, in women with completed childbearing who have extensive uterine involvement, heavy bleeding, or additional risk factors for persistent GTN—after thorough counselling and shared decision-making. Although hysterectomy reduces the risk of GTN, it does not prevent all cases of metastatic disease [30]. Postoperative monitoring of hCG levels and reliable contraception for 1 year is essential.

A rarity of hydatidiform mole pregnancies are so-called twin/multiple pregnancies with complete/partial hydatidiform moles (complete hydatidiform moles = CHM, partial hydatidiform moles = PHM) and coexisting fetus or coexisting fetuses (coexisting fetus = CF) [31]. There are over 200 case reports in the literature worldwide, mainly from Asia [32]. The incidence of these pregnancies (hydatidiform mole and coexisting fetus = HMCF) has been reported to be between 1:20,000 and 1:100,000 [33]. The majority of these are complete moles (CHMCF) and, less commonly, partial moles (PHMCF) [34]. The former are particularly associated with maternal complications such as vaginal bleeding, intrauterine fetal death (IUFD), preterm birth, pre-eclampsia, persistent gestational trophoblastic disease (pGTD) [35].

To date, only five cases similar to our case study have been described in the literature, with the occurrence of cardiorespiratory symptoms due to deterioration of LV function in previously heart-healthy women shortly before or after induction of abortion in PHM or CHMCF, mostly in the second trimester of pregnancy [35–39]. The cases had a significantly elevated serum hCG concentration and a rapid recovery or improvement of LV function in the short term in common.

Unlike peripartum cardiomyopathy, the onset of (acute) heart failure did not occur in the last month of pregnancy, during labor or in the first postpartum months [40], but early in pregnancy. Another unique feature is the presence of a hydatidiform mole. Despite these differences, the clinical course is similar to the clinical course peripartum cardiomyopathy (PPCM).

Peripartum cardiomyopathy is defined as a deterioration in systolic left ventricular function (LVEF) < 45% in previously heart-healthy women in addition to the abovementioned time of occurrence [41]. Possible pre-existing conditions with similar symptoms such as dilated cardiomyopathy, familial cardiomyopathy, Takotsubo cardiomyopathy, myocarditis, valvular heart disease, or congenital heart disease should therefore be excluded first [40–44].

Clinically, the symptoms of heart failure can be very variable in patients, ranging from fatigue, palpitations, and persistent cough to dyspnea/orthopnea, reduced exercise tolerance, peripheral edema, and cardiogenic shock. As the symptoms may mimic physiological changes during or after pregnancy, as well as pregnancy complications such as pulmonary embolism, amniotic fluid embolism, or pregnancy-related myocardial infarction, there is a risk of delay in diagnosis and initiation of treatment [45, 46].

Known risk factors for developing PPCM include advanced age, subfertility, previous malignancy, ethnicity or genetic predisposition, multiparity, multiple pregnancies, and pre-eclampsia [47–50].

The exact etiopathogenesis of PPCM in humans is not fully understood, but current research suggests a “multiple hit hypothesis” in which pregnancy hormones, angiogenesis, and cardiomyocyte metabolism appear to play a central role in the development of PPCM [48]. In two transgenic mouse models with gene knock-out of STAT-3 or PGC-1*α*, increased oxidative stress resulted in increased cleavage of the lactogenic hormone prolactin to a cytotoxic 16 kDa fragment, which primarily affected endothelial cells and secondarily impaired myocardial metabolism via endothelial damage. Inhibition of prolactin release using bromocriptine, a dopamine D2 receptor agonist, reduced or prevented the occurrence of PPCM in the mice [51, 52].

If PPCM is suspected on the basis of the above-described symptoms, a diagnostic algorithm consisting of ECG, laboratory determination of NT-proBNP, chest X-ray, and TTE is recommended [41]. A PPCM-specific ECG pattern has not yet been identified, but standard ECG findings in patients are rather rare. The most common ECG pathology documented in PPCM patients is an alteration of the T wave, particularly T inversion, which accounts for about 70% of patients [53]. In terms of laboratory chemistry, patients usually have an elevated NT-proBNP with BNP > 100 pg/mL or NT-proBNP > 300 pg/mL. Echocardiography can confirm an LVEF < 45% and rule out possible differential diagnoses. Based on the abovementioned parameters, PPCM can be further classified into mild, moderate, and severe forms. This will determine the further therapeutic approach [41]. The so-called BOARD concept is generally recommended, consisting of bromocriptine, oral heart failure medication, anticoagulation, vasorelaxants, and diuretics. In particular, treatment with bromocriptine and anticoagulants should be applicated for all degrees of PPCM severity [54]. The use of bromocriptine not only improved the outcome or recovery of pump function in isolated left ventricular impairment, but also in the presence of additional right ventricular deterioration [55]. In addition, PPCM patients who received bromocriptine immediately after birth had a better outcome in subsequent pregnancy than those who did not receive bromocriptine treatment [56]. If treatment with bromocriptine is not possible, cabergoline, a selective dopamine D2 receptor agonist, may be a good alternative, according to the results of recent studies [57]. Worldwide, mortality rates for PPCM range from 2% to 30% [40].

The management of cardiogenic shock in PPCM can be challenging. For example, treatment with typical *β*-adrenergic stimulants such as dobutamine is associated with a worse cardiac outcome in PPCM [58]. Treatment with levosimendan did not improve outcome [59]. In the mouse model, co-medication with perhexiline showed a reduction in ß-adrenergic cardiotoxicity [60]. In refractory cardiogenic shock, the use of mechanically active circulatory support systems such as Impella [61], or LVAD [62], in addition to optimal intensive care and bromocriptine therapy, has been shown to be effective.

Early after diagnosis, patients with severely impaired LV function were at increased risk of ventricular tachyarrhythmias [63]. The use of portable defibrillators has been shown to be a safe, noninvasive, and effective option to prevent sudden cardiac death with a high recovery rate from PPCM [64]. Encouragingly, PPCM has a high recovery rate of LV function of 60% at 6 months [65] and 72% at 12 months [66]. A wearing period of 3 to 6 months has been recommended [64].

Specific biomarkers for diagnosis, prognosis, or treatment monitoring are not yet available. Angiogenesis factors such as copeptin, sFlt-1, PIGF, and cathepsin D are potential candidates [67].

Regular follow-up is recommended, not only to assess LV function, heart failure symptoms, and therapy, but also for PPCM-associated arterial hypertension, arrhythmias, thromboembolism, thyroid dysfunction, and increased tumor risk [68–70].

This case is unique due to the rare occurrence of a partial hydatidiform mole complicated by severe pregnancy-associated cardiomyopathy. While partial hydatidiform moles are often misdiagnosed as incomplete abortions and typically associated with early fetal demise, the development of acute, severe biventricular heart failure in a previously healthy young woman is exceptionally uncommon. To date, only a handful of similar cases have been reported, all describing significant cardiac dysfunction around the time of abortion induction in the second trimester.

The case highlights the critical importance of early recognition and multidisciplinary management of this rare but life-threatening complication. The combination of trophoblastic disease with severe cardiac impairment demands close cardiological and gynecological follow-up to prevent long-term morbidity. This report adds valuable insight into the clinical course, diagnostic challenges, and therapeutic strategies needed to manage such complex presentations effectively.

## 2. Case Report

The 19-year-old patient was transferred to the Hannover Medical School in her 18th week of pregnancy for further management of a hydatidiform mole and late abortion. She was previously known to have undergone three appendectomies with subsequent revision for abscess formation in 02/2022.

The patient reported experiencing progressive dyspnea and a dry cough beginning on September 19, without accompanying fever or other systemic symptoms. A COVID-19 PCR test performed externally was negative.

On September 27, during a gynecological consultation, the absence of fetal cardiac activity was noted via ultrasound. Due to the respiratory symptoms reported during this visit, she was referred to the local hospital, where an intrauterine fetal demise (IUFD) was confirmed. Additionally, the ultrasound findings raised suspicion of a molar pregnancy. The patient was subsequently transferred to our tertiary care center for further management.

At presentation to our center, the patient reported not having been aware of her pregnancy until recently. The initial diagnosis of pregnancy and molar pregnancy was made externally prior to referral. On clinical examination, the uterus was found to be enlarged, corresponding to approximately 18 weeks of gestation. No fetal heart sounds were detectable. Ultrasound examination confirmed the presence of a hydatidiform mole.

On clinical examination, the patient was dyspneic with peripherally reduced oxygen partial saturation, in a markedly decreased general condition with hypertension and tachycardia with normal nutritional status. In addition, the patient had been suffering from progressive dyspnea, dry cough without sputum, and abdominal pain for 1 week. On transvaginal ultrasound at admission, the uterus appeared enlarged and contained a voluminous, grape-like, multicystic placental mass suggestive of a molar pregnancy. Adjacent to the mass, a fetus without detectable cardiac activity was identified, consistent with intrauterine fetal demise (IUFD). These findings raised suspicion of a partial hydatidiform mole, which was later confirmed by histopathological analysis.

In a case of hCG-induced latent hyperthyroidism with suppressed TSH and normal peripheral hormones, treatment with propranolol 10 mg 1x/day was prescribed for sinus tachycardia and hypertension. With increasing dyspnea, a chest X-ray was performed, showing cardiomegaly with pulmonary congestion. Pneumothorax and pleural effusion were excluded.

On the same day, medical abortion was initiated. After induction of abortion with mifepristone 200 *μ*g, followed by misoprostol 400 *μ*g, a slight opening of the cervix and irregular contractions resulted in excessive bleeding with tissue leakage. Sonographically, both the fetus and significant remnants of the intrauterine cystic tissue were still visible, necessitating urgent suction evacuation followed by post-curettage under ultrasound guidance. The surgery was uneventful. With an intraoperative blood loss of approximately 1.5 L, intraoperative administration of two erythrocyte concentrates was indicated. Postoperatively, the patient was stable at all times and was monitored in the delivery room for 6 h.

**Figure 1:**
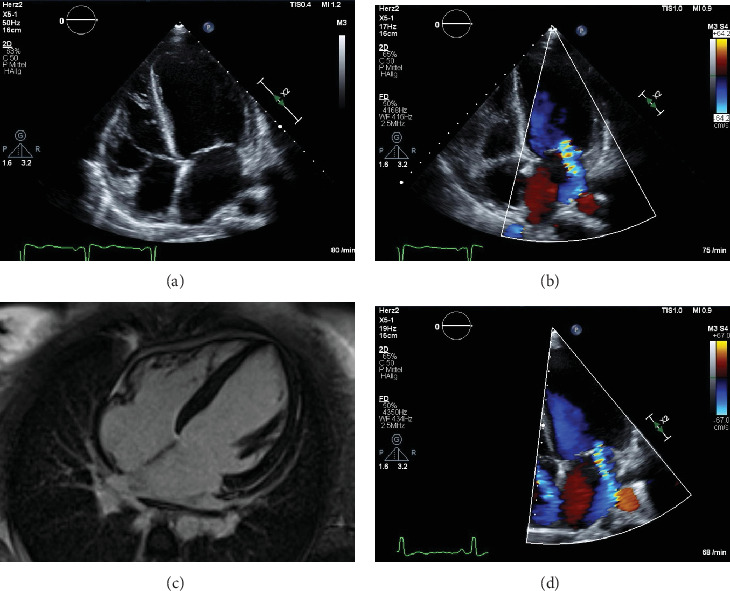
(A) Ballooning of LV and RV in four-chamber view in TTE at admission. (B) Mitral regurgitation in four-chamber view in TTE at admission. (C) Four-chamber view at CMR before discharge. (D) Mitral regurgitation in four-chamber view in TTE before discharge.

Post-operative transthoracic echocardiography revealed severely impaired left ventricular (LV) function, reduced right ventricular (RV) function and severe mitral valve regurgitation with sinus tachycardia (see Figure 1a,b). On suspicion of pregnancy-associated cardiomyopathy (PACM), the patient was immediately transferred to the cardiac intensive care unit. At admission, the patient was respiratory and hemodynamically stable. Hemodynamic monitoring was performed with a Swan-Ganz catheter.

Laboratory chemistry showed a marked increase in NT-proBNP (> 35,000 ng/L) and prolactin (521 ng/mL), suggesting a pregnancy-induced cardiomyopathy similar to peripartum cardiomyopathy. Treatment for heart failure was started with enalapril, bisoprolol, eplerenone, empagliflozin, and torasemide, as well as bromocriptine with thromboprophylaxis with low-molecular-weight heparin.

**Figure 2:**
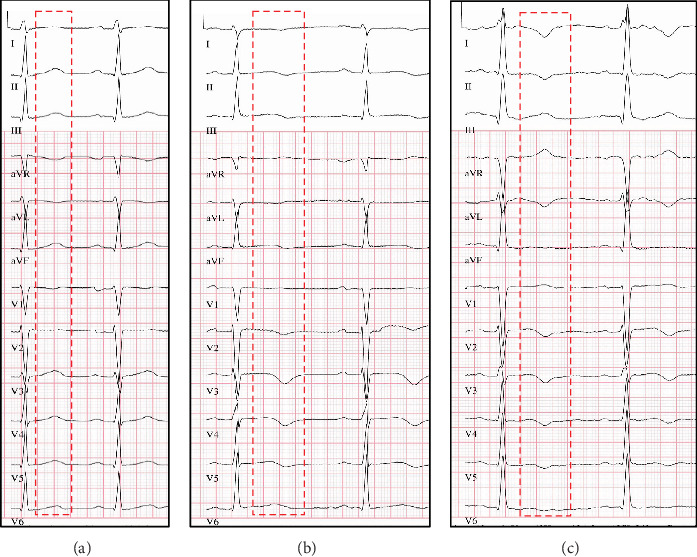
T-wave abnormalities over time (50 mm/s, 10 mm/mV). (a) ECG at admission: normal T-waves. (b) ECG at ICU: inverted and biphasic T-waves. (c) ECG before discharge: transition from biphasic to inverted T-waves.

The ECG showed normal sinus rhythm with a new biphasic T wave in II/III/aVF/V4/V6 and a new concordant T-neg in V2/V3 (see Figure 2).

At admission, the patient also had acute renal failure (AKIN II) with mild hyperkalemia and metabolic acidosis, which resolved with differentiated volume and diuretic administration.

In the case of fever, rising laboratory parameters of inflammation, cough with sputum production and radiologically confirmed congestion, a calculated antibiotic therapy with piperacillin/tazobactam and azithromycin was initiated in the case of congestive pneumonia. The values decreased during this treatment.

After induction of abortion and under the initiated therapy, there was an increasing improvement in LV function in the echocardiographic controls from approximately 31% initially to up to 47% at the time of discharge.

**Figure 3:**
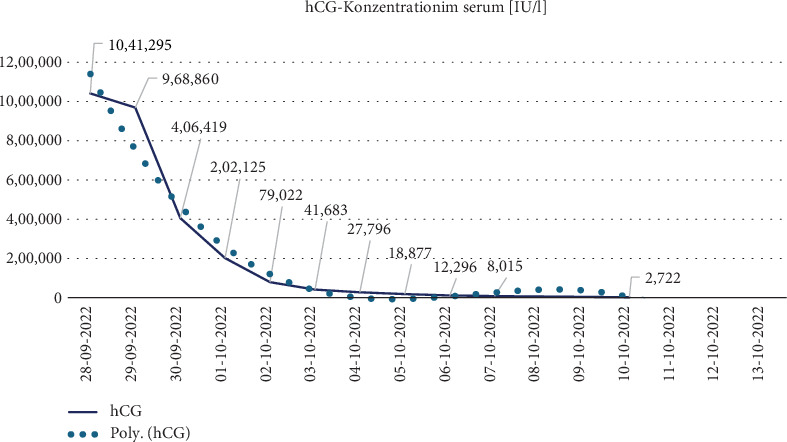
Timeline of hCG serum concentrations (IU/L).

A resting cardiac MRI scan showed no evidence of differential diagnosable diseases. Subsequently, prolactin, hCG, and TSH levels normalized and there was a complete recovery of LV function (see Figure 1c), although mitral regurgitation persisted (see Figure 1d). The course of hCG levels is shown graphically in Figure 3.

**Figure 4:**
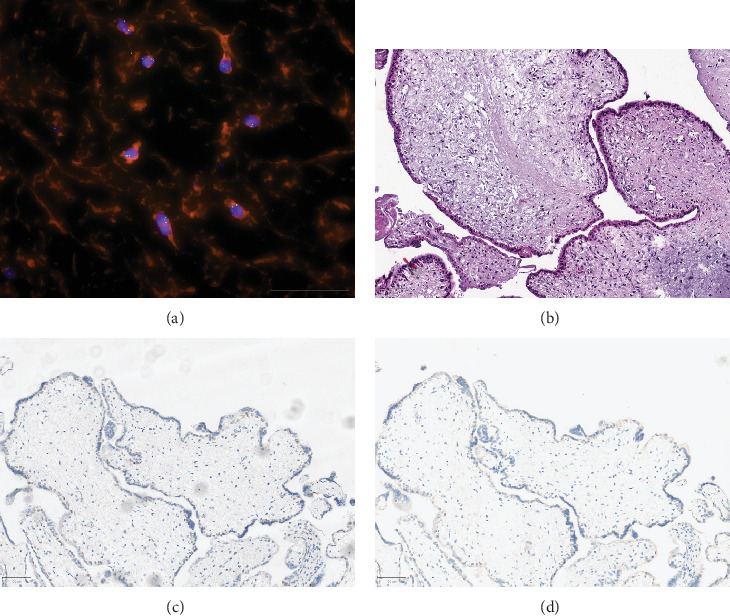
Histological stainings of the placenta. (a) FISH, 60-fold magnification, detection of X-chromosome triploidy in cytotrophoblastic cells. (b) Hematoxylin–eosin staining, 100-fold magnification. (c) p57 staining, 100-fold magnification, distinct nuclear staining of several cytotrophoblastic cells and of a small amount of placental villous stroma cells. (d) p63 staining, 100-fold magnification, weak nuclear staining of few cytotrophoblastic cells.

Based on the histological examination of the fetal tissue, the histological findings, the immunohistochemistry, and the trisomy of the X chromosome detected by fluorescence in situ hybridization, the diagnosis was a partial hydatidiform mole (Figure 4).

To clarify the diagnostic process and to systematically address potential differential diagnoses, we have summarized the relevant clinical findings, laboratory results, and imaging studies in Table 1. This table outlines the key considerations and the rationale behind the exclusion or confirmation of each differential diagnosis, including thyroid storm, trophoblastic embolization, and cardiac events secondary to intrauterine fetal demise.

The patient was discharged after 15 days in good subjective health.

To compare our case with the existing literature, we summarized similar reported cases of molar pregnancy associated with cardiac complications in Table 2 [35–39]. These reports consistently underline the importance of early diagnosis, individualized management, and close follow-up for both cardiac and gynecological outcomes.

Following discharge, close follow-up was recommended, including regular outpatient visits to both the gynecological and cardiological clinics. Reliable contraception for a duration of 12 months was advised. Serial measurements of *β*-hCG demonstrated a continuous decline, reaching undetectable levels by 15 November 2022. Although initial cardiological follow-up was initiated, further assessment of cardiac function and valvular status was unfortunately limited, as the patient did not attend the scheduled appointments. Nevertheless, the importance of continued monitoring was emphasized to the patient due to the risk of persistent gestational trophoblastic disease and potential cardiac complications.

## 3. Discussion

Consistent with PPCM, our patient presented with symptoms of heart failure with cough and dyspnea, laboratory tests showed significantly elevated nt-proBNP, chest X-ray showed cardiopulmonary congestion, and transthoracic echocardiography showed new onset of severely impaired LV function. ECG changes and acute renal failure, indicative of acute deterioration in LV function, occurred only during hospitalization. Laboratory chemistry also showed significantly elevated prolactin.

However, in contrast to classic PPCM, the above findings occurred at the beginning of the second trimester with intrauterine fetal death and not at the end of the pregnancy, suggesting pregnancy-associated cardiomyopathy (PACM). Several cases of this have been reported in the literature, in rarer cases as early as the 17th week of pregnancy [71].

In our case report, the unusually late presentation in her 18th week of pregnancy raises the question of whether earlier recognition and intervention might have prevented the cardiac complications observed. In this case, the diagnosis of the molar pregnancy was delayed until the second trimester, which may have contributed to the development of cardiac complications. The patient had not been aware of the pregnancy for a significant period, which led to a lack of early prenatal care and imaging. This highlights how unrecognized pregnancies—particularly in patients with limited early symptom awareness or delayed healthcare access—can result in missed opportunities for early diagnosis and intervention. Increased clinical vigilance and timely assessment remain essential to prevent severe sequelae in such cases.

Tachymyopathy due to hCG-induced hyperthyroidism with documented tachycardia should be considered in the differential diagnosis. In the referring hospital, heart rates between 100 and 115 beats/min were measured after diagnosis, and in our monitoring, a maximum heart rate of 106 beats/min with sinus tachycardia was measured during admission to the cardiac intensive care unit. It is unclear how long the ß-hCG-induced hyperthyroidism or tachycardia had been present and what heart rate and rhythm the patient had at home. Hyperthyroidism has been described in the literature to occur as early as the 11th week of pregnancy in hydatidiform moles [72]. Unfortunately, clear diagnostic criteria for tachymyopathy are not yet available, but sinus tachycardia seems to be less commonly associated with tachymyopathy [73].

In addition, based on the fulfilment of 3/4 of the revised Mayo Clinic criteria [74] and 4/8 of the InterTAK diagnostic criteria [75] with moderate troponin/CK-MB and significant NT-proBNP elevation, the new T-wave change, the transient worsening of LV function, the exclusion of myocarditis/MINOCA, the absence of pheochromocytoma or coronary artery disease, and the presence of a physical and psychological trigger, IUFD could be considered in partial moles with hyperthyroidism. Physical and psychological triggers for IUFD in partial moles with hyperthyroidism. However, age [75] and the presence of global hypokinesia are atypical for takotsubo cardiomyopathy [74, 75].

It remains unclear whether or not the presence of a hydatidiform mole plays a role in the development or prevention of PPCM or PACM. Two cases of PHMCF have been described in Japan, one case of CHM in the United States and, together with our case, three cases of PHM in Switzerland, the United States, and Germany.

The estimated incidence of PPCM is 1:300 to 1:100 pregnancies in endemic areas such as Nigeria or Haiti [76], 1:1000 in South Africa [77], 1:1500 in the United States [76, 78] and Germany [41], 1:10,000 in Denmark [79], and 1:15,500 in Japan [80].

There are also regional variations in molar pregnancies. An incidence of approximately 1 to 2:1000 pregnancies is reported for China/Japan; 12:1000 for Indonesia, India, and Turkey; and 0.5 to 1:1,000 for North America, Europe, and Oceania [81]. The ratio of PHM to CHM varies not only regionally [82], but also according to the age of the pregnant women [83]. HMCF has been described with an incidence of 1:20,000 to 1:100,000, with approximately 200 cases to date, including approximately 40 cases of PHMCF. An increased risk of PPCM or PACM therefore seems more likely in PHMCF than in CHM and PHM.

It should be noted that hydatidiform moles are more common in very young or older women (≤ 15 years, ≥ 45 years) [9] and PPCM in older women [84]. Although partial moles are often underdiagnosed [85], they are often misdiagnosed as missed or incomplete miscarriages in the first trimester due to the lack of sonographic assessment at this stage [86, 87]. This is therefore a stage of pregnancy well before the time of diagnosis of a classic PPCM or PACM.

With regard to prolactin, similar or even lower serum levels were found when comparing molar pregnancies [88, 89]. In patients with pGTD, prolactin levels were significantly higher than in patients before/after molar pregnancy and without pGTD [88]. Studies measuring 16-kDa prolactin, PIGF, copeptin, or vasoinhibins in molar pregnancies have not yet been published.

Elevated sFlt-1 levels have been described in molar pregnancies [90, 91], similar to pre-eclampsia [92] and PPCM [52]. Molar pregnancies are known for an early onset of pre-eclampsia between 15 and 20 weeks' gestation and the occurrence of complications from 32 weeks' gestation [93].

An older study from the 1990s provides immunohistochemical evidence of increased cathepsin D in CHM [94].

Even if a coincidence of the two rather rare diseases, hydatidiform mole and PPCM, seems rather unlikely due to their typical temporal occurrence during pregnancy, there are some similarities, such as occurrence at an older age, increased risk of pre-eclampsia, increased subfertility and increased sFlt-1.

### 3.1. Limitations

It was not possible to assess possible malformations due to the severe laceration and probably also due to the IUFD that had been present for some time. There was no sonographic evidence of malformations. There were also limitations in the karyotyping of the fetus. Based on the histological findings, the immunohistochemistry, and the trisomy of the X chromosome detected by fluorescence in situ hybridization, a partial mole was suspected. Additional karyotyping would have been necessary to confirm the diagnosis and exclude the rare PHMCF.

SNP analysis could also have been used to elucidate the genesis of paternal triploidy, as partial moles can in principle arise either from oocyte dispermy or from a diploid spermatozoon as a result of complete non-disjunction during spermatogenesis (meiosis II) [87, 95, 96]. However, more recent data have shown that the majority of paternal triploidies result from dispermy and rarely from diploid spermatozoa [95, 96]. It remains unclear why not all parental triploidies lead to the development of hydatidiform moles [97].

Due to the lack of data on PIGF, 16-kDa prolactin, cathepsin D, copeptin, and vasoinhibin in moles, it would have been very interesting to determine the above biomarkers. Unfortunately, the markers were not determined.

### 3.2. Strength

This case report's strength is its detailed presentation of a very rare and severe complication, pregnancy-associated cardiomyopathy, in a patient with a partial hydatidiform mole, thereby contributing valuable insight to the limited existing literature. Notably, the report includes a comprehensive multidisciplinary diagnostic approach, incorporating imaging, histopathology, immunohistochemistry, fluorescence in situ hybridization (FISH), echocardiography, and laboratory markers. The stepwise management from medical abortion to intensive cardiac care and heart failure treatment is clearly described, offering practical guidance. The documentation of follow-up data showing clinical improvement and normalization of biochemical parameters emphasizes the importance of close monitoring. Finally, placing the case in the context of previously reported cases enhances understanding and awareness of this rare but serious complication.

## 4. Conclusion

In the case of a hydatidiform mole as a benign trophoblastic tumor, early diagnosis is essential to prevent serious symptoms in the course of the disease. In particular, if cardiac symptoms occur, early diagnosis should be carried out by ECG, TTE, and laboratory determination of nt-proBNP. Close cardiological and gynecological follow-up is particularly important in these patients. Because of the parallels between molar disease and PPCM, further investigations, including molecular markers, should be carried out in the future.

### 4.1. “Take-Away” Lessons

This case highlights the importance of early recognition and diagnosis of partial hydatidiform mole, especially when complicated by rare but severe pregnancy-associated cardiomyopathy. It underscores the need for a multidisciplinary approach involving gynecologists and cardiologists to manage both the trophoblastic disease and the cardiac complications effectively. Close monitoring and follow-up are essential to ensure recovery and prevent long-term sequelae. Awareness of this rare presentation can improve timely intervention and patient outcomes in similar cases.

## Figures and Tables

**Table 1:** Differential diagnoses considered with corresponding clinical findings, investigations, and rationale for exclusion or confirmation.

**Differential diagnosis**	**Clinical findings**	**Investigations**	**Outcome/reasoning**
Thyroid storm	Fever, tachycardia, agitation, altered mental status	Labs: TSH, free T3, free T4; clinical evaluation	Slightly suppressed TSH, normal free T3 and T4, no clinical signs of thyroid storm; **excluded**
Trophoblastic embolization	Sudden clinical deterioration, pulmonary symptoms	Chest CT, ultrasound, clinical monitoring	No radiological evidence or clinical signs; **excluded**
Intrauterine fetal death (IUFD)	Absence of fetal heartbeat, clinical presentation	Ultrasound	IUFD confirmed, but not directly causing cardiac event
Cardiac failure due to other causes	Dyspnea, edema, heart murmurs	Echocardiography, NT-proBNP, other labs	Cardiac dysfunction confirmed; **other causes ruled out**
Molar pregnancy with complications	Grape-like placental tissue, elevated *β*-hCG	Ultrasound, *β*-hCG measurement	Diagnosis confirmed and considered causative pathology

**Table 2:** Summary of comparable cases of hydatidiform mole and cardiac involvement including clinical and gynecological outcomes.

**Author (year)**	**Type of mole/pregnancy**	**Gestational age**	**Cardiac presentation**	**Management**	**Cardiac outcome**	**Gynecological outcome**
Moriuchi et al. (2017) [35]	CHM + coexisting fetus	12 weeks	Hypertension and acute heart failure	Termination + HF treatment	Recovery	Development of GTD
Massad et al. (1993) [36]	CHM	12 weeks	Congestive heart failure	Termination + HF treatment	Recovery	No details on trophoblastic follow-up
Saito et al. (2015) [37]	CHM + fetus viable	11 weeks	Hypertension, cardiac enlargement, PACM, worsened post-abortion	Termination + HF treatment	Recovery	No details on trophoblastic follow-up
Billieux et al. (2004) [38]	PHM fetus viable	16 weeks	Hypertension, pre-eclampsia, postpartal congestive heart failure	Termination + HF therapy	Recovery	No GTN; authors discussed potential genetic factors
Cao et al. (2022) [39]	PHM	17 weeks	Heart failure	Termination + HF therapy	Recovery	No progression to GTN

## Data Availability

Data were generated at the Department of Obstetrics and Gynecology of Hannover Medical School (MHH). The datasets used and analyzed during the current study are available from the corresponding author upon reasonable request.

## Supporting Information

Supporting Information Additional supporting information can be found online in the Supporting Information section. The file CARE-checklist–Case Report.pdf has been included as supplementary material. This document provides a detailed checklist following the CARE (CAse REport) guidelines, ensuring completeness and transparency in the reporting of this case.
